# Kn-Ba: a novel serine protease isolated from *Bitis arietans* snake venom with fibrinogenolytic and kinin-releasing activities

**DOI:** 10.1186/s40409-018-0176-5

**Published:** 2018-12-13

**Authors:** Ângela Alice Amadeu Megale, Fábio Carlos Magnoli, Alexandre Kazuo Kuniyoshi, Leo Kei Iwai, Denise V. Tambourgi, Fernanda C. V. Portaro, Wilmar Dias da Silva

**Affiliations:** 1 Immunochemistry Laboratory, Butantan Institute, São Paulo, 05503-900 Brazil; 2 Special Laboratory of Applied Toxinology / Center of Toxins, Immune-Response and Cell Signaling (CeTICS), Butantan Institute, São Paulo, 05503-900 Brazil

**Keywords:** *Bitis arietans*, Venom, Antivenom, Serine protease, Fibrinogenolytic, Kinin-releasing activity

## Abstract

**Background:**

*Bitis arietans* is a venomous snake found in sub-Saharan Africa and in parts of Morocco and Saudi Arabia. The envenomation is characterized by local and systemic reactions including pain, blistering, edema and tissue damage, besides hemostatic and cardiovascular disturbances, which can cause death or permanent disabilities in its victims. However, the action mechanisms that provoke these effects remain poorly understood, especially the activities of purified venom components. Therefore, in order to elucidate the molecular mechanisms that make the *Bitis arietans* venom so potent and harmful to human beings, this study reports the isolation and biochemical characterization of a snake venom serine protease (SVSP).

**Methods:**

Solubilized venom was fractionated by molecular exclusion chromatography and the proteolytic activity was determined using fluorescent substrates. The peaks that showed serine protease activity were determined by blocking the proteolytic activity with site-directed inhibitors. In sequence, the fraction of interest was submitted to another cycle of molecular exclusion chromatography. The purified serine protease was identified by mass spectrometry and characterized biochemically and immunochemically.

**Results:**

A serine protease of 33 kDa with fibrinogen-degrading and kinin-releasing activities was isolated, described, and designated herein as Kn-Ba. The experimental Butantan Institute antivenom produced against *Bitis arietans* venom inhibited the Kn-Ba activity.

**Conclusions:**

The in vitro activities of Kn-Ba can be correlated with the capacity of the venom to provoke bleeding and clotting disorders as well as hypotension, which are common symptoms presented by envenomed victims. Obtaining satisfactory Kn-Ba inhibition through the experimental antivenom is important, given the WHO’s recommendation of immunotherapy in cases of human accidents with venomous snakes.

**Electronic supplementary material:**

The online version of this article (10.1186/s40409-018-0176-5) contains supplementary material, which is available to authorized users.

## Background

Snakebite is a serious and neglected public health problem worldwide, affecting mainly developing countries and more specifically the rural areas. In sub-Saharan Africa an estimated 90,000–400,000 envenoming snakebites occur every year, resulting in up to 32,000 deaths [1] and 14,000 victims suffering amputations, local tissue damage and chronic disabilities [2]. However, these data are probably underestimated because they are based on just a few case reports or on the epidemiological literature.

Snake venoms are a complex mixture of different toxins, which have a wide range of physiological effects; among them, snake venom serine proteases (SVSPs) constitute one of the most important components [3, 4]. Serine proteases from snake venoms are classified into clan PA, S1 family of chymotrypsin, and present the catalytic triad (His43, Asp88 and Ser184, chymotrypsin numbering) highly conserved [5]. Thus, it is widely known that venom serine proteases exhibit remarkable resistance to inhibition by human serine protease inhibitors, the serpins [6]. In contrast to trypsin, the SVSPs are characterized by high specificity for their substrates, despite which they present a high degree of amino-acid sequence identity with each other. Typically, SVSPs exhibit about 51–98% identity with each other, 26–33% with human thrombin and 34–40% with human plasma kallikrein [7].

In general, the SVSPs affect the coagulation cascade through the activation of components involved in the coagulation, fibrinolysis and platelet aggregation processes through mechanisms that mimic mammalian enzymes. Some SVSPs, by simulating the action of thrombin, have been termed thrombin-like enzymes (TLE) and are present in various snake venoms. Usually, they are single-chain serine proteases and have relative molecular mass ranging from 26 to 33 kDa, depending on the degree of glycosylation [7]. Some examples of thrombin-like snake serine proteases are batroxobin [8, 9] and TL-BJ [10], which act upon fibrinogen and may lead to hemostatic imbalance in envenomed victims and preys. Interestingly, while some SVSPs can degrade fibrinogen, leading to the formation of fibrin clots [11], others, like halystase, can cleave fibrinogen at different thrombin sites, without inducing fibrin clotting [12]. In contrast, rhinocerase, isolated from the *Bitis gabonica rhinoceros* venom, has the ability to completely dissolve plasma clots generated by thrombin, suggesting that this SVSP might present fibrin-degrading rather than coagulating activity [13].

Some SVSPs are able to release bradykinin (BK) or kallidin (Lys-BK) through kininogen hydrolysis, such as crotalase [14, 15], elegaxobin II [11, 16] and KN-BJ [17]. These serine proteases are known as *kallikrein-like enzymes*. In particular, the effects of bradykinin have been well described and are particularly active on vascular musculature, resulting in vasodilation and increased vascular permeability [18, 19]. Thus, the *kallikrein-like enzymes* can be considered important molecules that lead the envenomed victim to hypotensive shock.

*Bitis arietans*, from the Viperidae family, is a venomous snake widely distributed throughout sub-Saharan Africa and in savannah and grasslands from Morocco and western Arabia [20, 21]*. B. arietans* (“puff adder”) is a common cause of serious envenoming and has been accused of causing more bites and deaths in humans and domestic animals than all the other African snakes put together [22]. Despite this, there have been very few clinical studies of patients with a proven puff-adder bite.

Proteomic analyses showed that metalloproteases, serine proteases, disintegrins, L-amino acid oxidase, Kunitz inhibitors, phospholipases A_2_, cystatins and C-type lectins are present in *Bitis arietans* venom [3, 23]. Until now, according to the literature, some toxins have been isolated from the *Bitis arietans* venom including: hemorrhagic [24–26], and non-hemorrhagic metalloproteases [27]; serine proteases with kinin-releasing and fibrinogenolytic activities [28], a fibrinogenase which is able to inhibit platelet aggregation [29]; phospholipases A_2_ like bitanarina which blocks ionic channels [30] and bitiscetina that induces platelet aggregation [31, 32]; a pro-coagulant C-type lectin-like [33]; and bitistatin [acession number: P17497], also known as arietin, that inhibited platelets aggregation [34]. Recently, a family of peptides from this venom with angiotensin-converting enzyme (ACE) inhibitory activity, the BPPs (Bradykinin-potentiating peptides), was described as presenting hypotensive activity in vivo [35]*.*

Human case reports show that a puff adder bite can lead to local and systemic effects. Local symptoms include swelling, pain, blistering, ecchymosis, necrosis and enlarged draining lymph nodes. The systemic effects can be related to fever, leukocytosis, hemostatic disturbances, hemorrhage, thrombocytopenia and hypotension; in the absence of antivenom treatment, the envenoming may be fatal [22, 36].

Based on the venom composition, as well as on the symptoms reported during the envenomation, the hypothesis of this study was that the venom of *Bitis arietans* contains several distinct proteases that cause hemorrhage and hypotension, although the purification and characterization of these proteases have not yet been fully accomplished. Therefore, to further understand the nature and the functions of isolated toxins, this present study reports the purification, partial amino-acid sequence and preliminary functional characterization of Kn-Ba, a SVSP isolated from *B. arietans* venom with fibrinogenolytic and kinin-releasing activities.

## Methods

### Venom

Lyophilized *B. arietans* venom was purchased from Venom Supplies, Tanunda, Australia. These venoms were obtained from males and females snakes of different ages, captured in South Africa and maintained in captivity. Stock solutions were prepared in sterile phosphate-buffered saline (PBS, 8.1 mM sodium phosphate, 1.5 mM potassium phosphate, 137 mM sodium chloride and 2.7 mM potassium chloride, pH 7.2) at 5 mg/mL, based on their protein concentration measured by the bicinchoninic acid method [37] using a Pierce BCA Protein Assay kit (Rockford, IL, USA), with bovine serum albumin as the standard protein.

### Antivenom

Experimental horse anti-*Bitis arietans* (α-Ba) antivenom, produced by Guidolin and collaborators [38], was kindly donated by the Antivenom Production Section of the Butantan Institute, São Paulo, Brazil. This antivenom, produced using *B. arietans* venom (Venom Supplies, Tanunda, Australia), was obtained from horse plasmas and purified by the caprylic acid method [39] and exhibited a high titer of 5.18 × 10^6^ U-E/mL [40]. Anti-botulinic F(ab’)_2_ fragments (batch n° 0908161; protein concentration of 48.9 mg/mL), kindly provided by the Butantan Institute, were used in this study as a negative control. The total protein content of used antibodies was determined by BCA assay.

### Purification of Kn-Ba

Venom was fractioned by molecular exclusion chromatography on a Superose 12 HR 10/30 column (Amersham Pharmacia Biotech AB, Uppsala, Sweden). All peak profiles were monitored by their absorbance at 280 nm using a UPC-900 monitor (Amersham Pharmacia Biotech AB). Briefly, in a climate-controlled room (22 ± 2 °C), 20 milligrams of venom was dissolved in five milliliters of column eluent and 500 μL was applied each time into the column, previously equilibrated with ammonium acetate 50 mM. In the same eluent, the proteins were eluted at a flow rate of 0.4 mL/min, and fractions were manually collected. Fraction 3 (3 mg/mL), obtained from gel filtration chromatography, was pooled and submitted to another cycle of molecular exclusion using a Superdex 75 10/300 GL column (GE Healthcare, Bio-Sciences AB, Uppsala, Sweden), following the abovementioned conditions. Proteins were freeze-dried, resuspended in sterile PBS and stored at − 20 °C. The protein content of the obtained fractions was estimated by BCA assay and the electrophoretic profile was visualized by SDS-PAGE [41] (4.0 μg/well resolved in 10% polyacrylamide gel) and silver-stained [42].

### Mass spectrometry analysis: Kn-Ba identification

Purified Kn-Ba, obtained after the last purification step, was subjected to an in-gel digestion with trypsin (Sigma-Aldrich, MO, USA) [43, 44]. The mixture was desalted by Zip-Tip, dried and then resuspended in 0.1% formic acid. Mass spectrometric analysis was performed by liquid chromatography in an Easy-nLC Proxeon nano-HPLC system coupled to an LTQ-Orbitrap Velos (Thermo Fisher Scientific, Bremen, Germany) through a nanoelectrospray ion source. The peptides were separated in a 10 cm column (75 μm × 350 μm) packed in-house with 5 μm Jupiter® C-18 beads (Phenomenex, Torrance, CA, USA). Peptides were eluted with a linear gradient of 5–95% acetonitrile, in 0.1% formic acid, in 15 min at a flow rate of 200 nL/min. Nanoelectrospray voltage was set to 2.1 kV, and the source temperature to 200 °C; the spectrometer was operated in data-dependent mode where the 5 most intense peaks were selected for collision-induced dissociation (CID) fragmentation after acquiring each full scan. The settings for the spectrometer were defined as: high-resolution full MS parameters (1 μscan; full MS mass range m/z of 200–2000 with a resolution of 60,000 and a target value of 1 × 10^4^ ions; max injection time = 100 ms). For fragment scans the settings were: isolation window of 2 Da, max list size of 500, exclusion duration time window of 15 s, a minimum signal of 5000, activation time = 10 ms and normalized collision energy = 35%. The raw data files were submitted to a search against the *“Serpentes”* database (taxid:8570) using PEAKS Studio (version 8, Bioinformatics Solution, Waterloo, Canada). A decoy database was also searched to calculate the False Discovery Rate (FDR) using the decoy-fusion method [45, 46]. The search parameters were: Trypsin digestion; precursor mass tolerance set to ±10 ppm and a fragment ion mass tolerance of ±0.5 Da; oxidized methionine (M + 15.99 Da) was set as variable modification and carbamidomethylation (C + 57.02) as fixed modification. The identified peptides were then sorted by their average of local confidence (ALC > 80%) to select the best spectra to annotate, and were filtered by FDR ≤ 5%.

### Proteolytic activity on FRET substrate: Inhibitors and antivenom effect

The proteinase activity assays were conducted in PBS (final volume 100 μL) using 96-well plates, and Fluorescent Resonance Energy Transfer (FRET) substrates at a final concentration of 10 μM (Abz-RPPGFSPFR-EDDnp and Abz-FRSSR-EDDnp, both provided by Dr. Luiz Juliano Neto, UNIFESP, Department of Biophysics). The reactions occurred at 37 °C and were initiated by the addition of 0.5 μg of *B. arietans* venom. The reactions were continuously monitored (fluorescence emission at 420 nm after excitation at 320 nm) in a fluorimeter (Victor 3™ Perkin-Elmer, Boston, MA, USA or FLUOstar® Omega, BMG Labtech, HE, Germany, according to the figure captions), as described by Kuniyoshi and collaborators [47]. Specific proteinase activity was expressed as units of free fluorescence of cleaved substrate *per* minute *per* μg of venom. There was an incubation period of 30 min at room temperature when PMSF (2 mM) and PHE (2 mM) were tested. The EDTA (100 mM) was used without pre-incubation time. When necessary, control samples were prepared in the presence of the same volume of ethanol used in the preparation of stock solutions of inhibitors (PMSF and PHE). The experiments were performed in quadruplicate.

The Abz-FRSSR-EDDnp substrate (10 μM) was used as a tool for the purification steps, since this FRET peptide, in the experiments described above, was the most selective for *B. arietans* SVSPs.

The ability of the experimental horse anti-*Bitis arietans* antivenom to neutralize the Kn-Ba proteolytic activity was estimated by incubating samples of purified serine protease, at room temperature for 30 min, in the presence or absence of different amounts of antivenom. The residual proteolytic activities of the venoms were measured as described above, using Abz-FRSSR-EDDnp as the substrate. The volume of the antivenom and the pre-incubation time for serum neutralization of the proteolytic activities were established to reach the maximum blocking effect of the Kn-Ba proteolytic activity. The experiments were performed in quadruplicate.

The specificity of anti-*Bitis arietans* antivenom (α-Ba antivenom) in neutralizing Kn-Ba was evaluated. Based on the amount of the *B. arietans* antivenom required to completely neutralize the Kn-Ba proteolytic activity, the neutralization control was accomplished using anti-botulinic F(ab’)_2_ fragments (α-botulinic serum). Kn-Ba residual proteolytic activity upon Abz-FRSSR-EDDnp substrate was assessed after incubation with α-botulinic serum at room temperature for 30 min and measured as described above.

### Cleavage of fibrinogen by Kn-Ba

Thirty micrograms of human fibrinogen (Sigma-Aldrich, MO, USA) was incubated with different concentrations of Kn-Ba (0.5 μg, 1 μg, 2 μg or 5 μg) for 1 h at 37 °C in a wet bath under constant gentle agitation. Next, samples were submitted to a 10% SDS-PAGE under reducing conditions and the gels were stained with Coomassie Brilliant Blue R-250. The fibrinogenolytic activity was determined by the cleavage of α, β and/or γ chains of the fibrinogen. The relative intensity of bands was estimated by densitometry (KODAK MI software).

### In vitro analysis of kinin-related peptides released by Kn-Ba

For determining kinin-related peptides released by Kn-Ba, 90 μM of kininogen-homologous peptide in the portion containing kinins, designated herein as KNBK (PLGMISLMKRPPGFSPFRSSR, GenOne, Rio de Janeiro, Brazil), was incubated with 0.2 μg of Kn-Ba in 50 mM Tris, 50 mM NaCl buffer, pH 7.4, at 37 °C for 3 h; the hydrolysis fragments were obtained by Zip-Tip and analyzed by mass spectrometry and described as follows. The raw data files were submitted to a search against the KNBK peptide sequence using PEAKS Studio (version 8, Bioinformatics Solution, Waterloo, Canada). A decoy database was also used. The search parameters were: no enzyme specificity; precursor mass tolerance set to ±10 ppm and a fragment ion mass tolerance of ±0.1 Da; oxidized methionine (M + 15.99 Da) was set as variable modification. The identified peptides were then sorted by their average of local confidence (ALC > 80%) to select the best spectra to annotate, and were filtered by FDR ≤ 0.1%.

### Statistical analysis

The data were expressed as mean ± standard error (SEM) and analyzed statistically using the software GraphPad Prism, version 5.1 for Windows (San Diego, USA). The statistical significance of results was calculated by One-Way analysis of variance (ANOVA) test followed by Tukey HSD post-hoc tests considering *p* values < 0.05 to be significant.

## Results

### FRET substrate screening

**Fig. 1:**
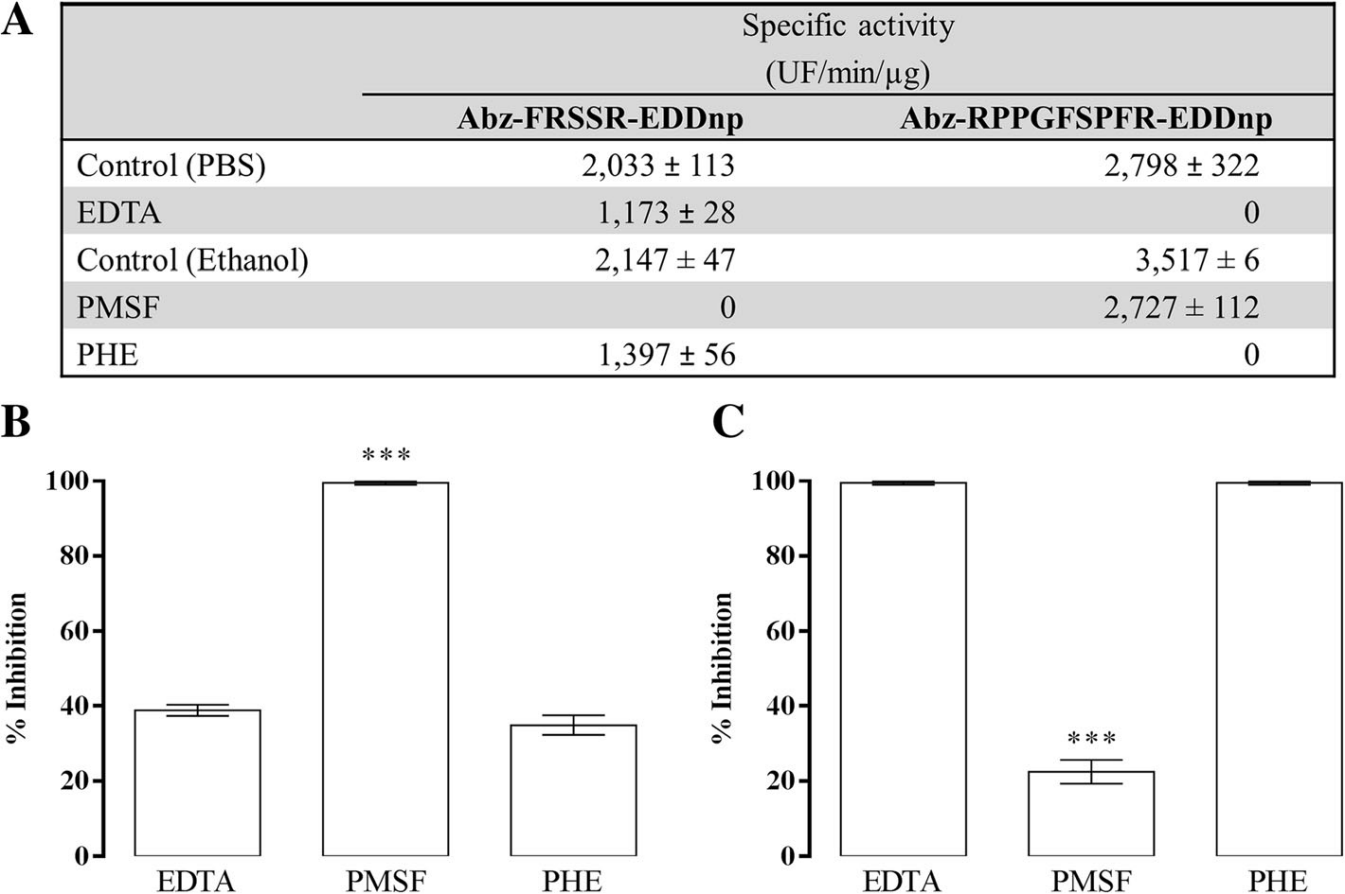
Venom proteolytic activity. **a** The proteolytic activity of venom, pretreated or not with EDTA (100 mM), PMSF (2 mM) and PHE (2 mM), was accessed by the hydrolysis of 10 μM of Abz-FRSSR-EDDnp and Abz-RPPGFSPFR-EDDnp FRETs substrates. The percentage of inhibition upon (**b**) Abz-FRSSR-EDDnp and (**c**) Abz-RPPGFSPFR-EDDnp substrates was determined. These assays were performed in quadruplicate. Results were expressed as specific activity (UF/min/μg of venom) ± SEM and analyzed statistically using One-Way ANOVA test followed by Tukey HSD post-hoc tests (*p < 0.05)

*B. arietans* venom presented proteolytic activity on both tested FRET substrates: Abz-FRSSR-EDDnp and Abz-RPPGFSPFR-EDDnp, which contain a sequence recognized by proteases of different catalytic natures or bradykinin amino acid sequence, respectively (Fig. 1). However, a complete activity inhibition by PMSF, which demonstrated mainly serine protease activity, was revealed when Abz-FRSSR-EDDnp substrate was used.

### Purification and identification of the Kn-Ba: A serine protease

**Fig. 2:**
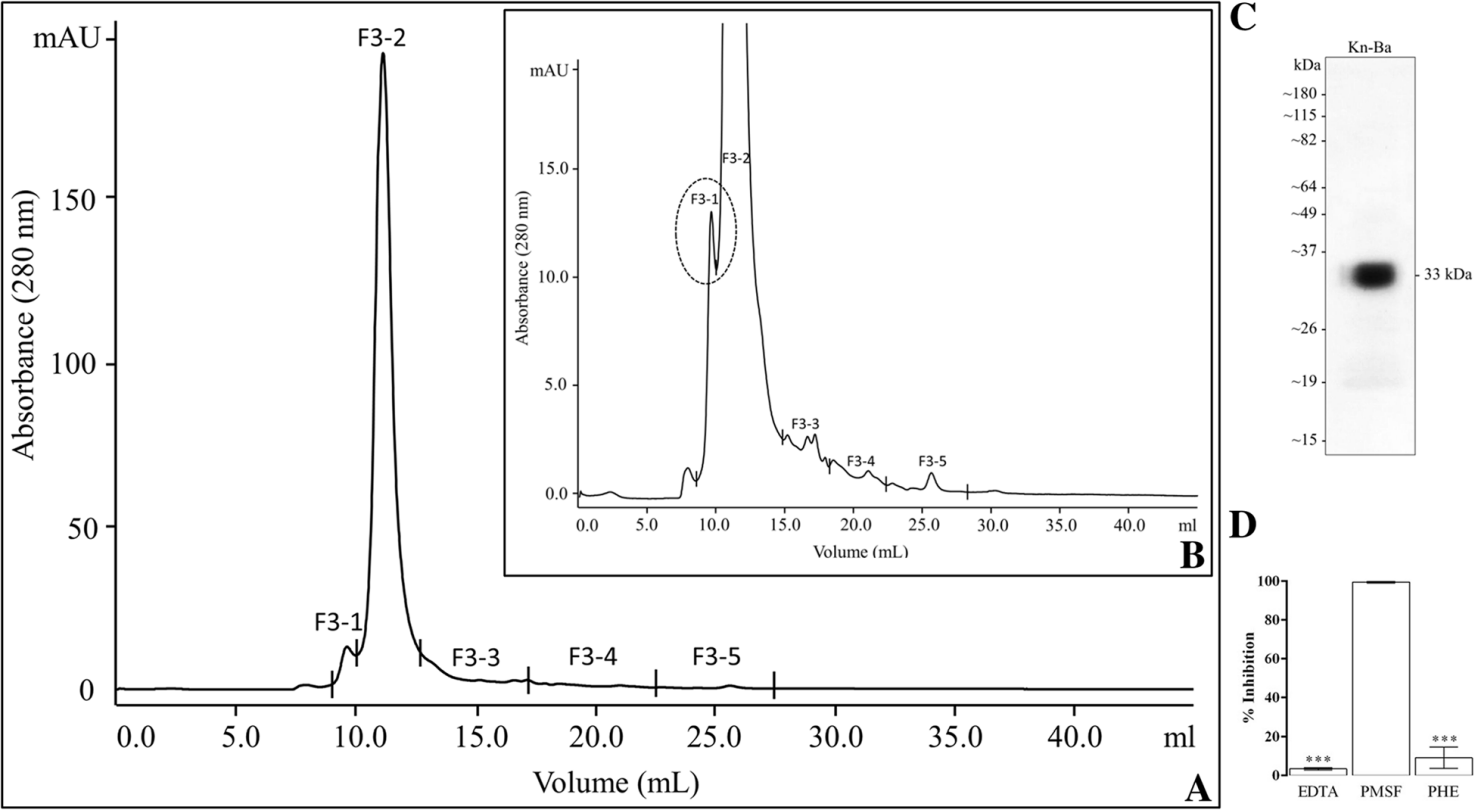
Purification and identification of Kn-Ba. **a** The third chromatographic peak (F3) obtained from the first purification step was subjected to another molecular exclusion chromatography on a Superdex 75 10/300 GL column, equilibrated and eluted with ammonium acetate 50 mM in a climate-controlled room (22 ± 2 °C). Samples were collected at a flow rate of 0.4 mL/min, and their protein content was monitored by recording the absorbance at 280 nm. **b** Zoom and highlight of F3–1, substrate-cleaving activity containing peak. **c** Silver-stained SDS-PAGE (10%) of the 33 kDa protein band corresponding to F3–1, which was denominated Kn-Ba. **d** The proteolytic activity of Kn-Ba, pretreated or not with EDTA, PMSF and PHE upon Abz-FRSSR-EDDnp FRET substrate. This assay was performed in quadruplicate. Results were expressed as inhibition percentage of proteolytic activity ± SEM and analyzed statistically using One-Way ANOVA test followed by Tukey HSD post-hoc tests (*p < 0.05)

*B. arietans* venom was fractionated using a Superose 12 HR 10/30 gel filtration column resulting in the elution of nine chromatographic peaks (Additional file 1). All fractions were tested using Abz-FRSSR-EDDnp FRET substrate; however, only F2 and F3 were able to cleave the substrate. The inhibition was performed using EDTA, PHE and PMSF, which are inhibitors of metallo- and serine proteases, respectively, and confirmed that both fractions contain serine proteases (Additional file 2). Fraction 2 presented a higher serine protease activity than fraction 3; however, the electrophoretic profile of F3 was less complex (Additional file 1). On the basis of these results, the third chromatographic peak was submitted to a second gel filtration step using a Superdex 7510/300 GL column. FRET substrate-cleaving activity was detected at peak 3–1 (F3–1, Fig. 2, panels a and b) and showed a single protein band of 33 kDa by SDS-PAGE (Fig. 2, panel c). Kn-Ba cleaved the substrate with high activity (2.374 ± 110), and, to assess and confirm the enzymatic nature of Kn-Ba, the assay was performed in the presence of PHE and EDTA or PMSF. The proteolytic activity of Kn-Ba was completely inhibited by PMSF, while PHE and EDTA had low effect, identifying Kn-Ba as a serine protease (Fig. 2, Panel d).

### Kn-Ba identification

**Fig. 3:**
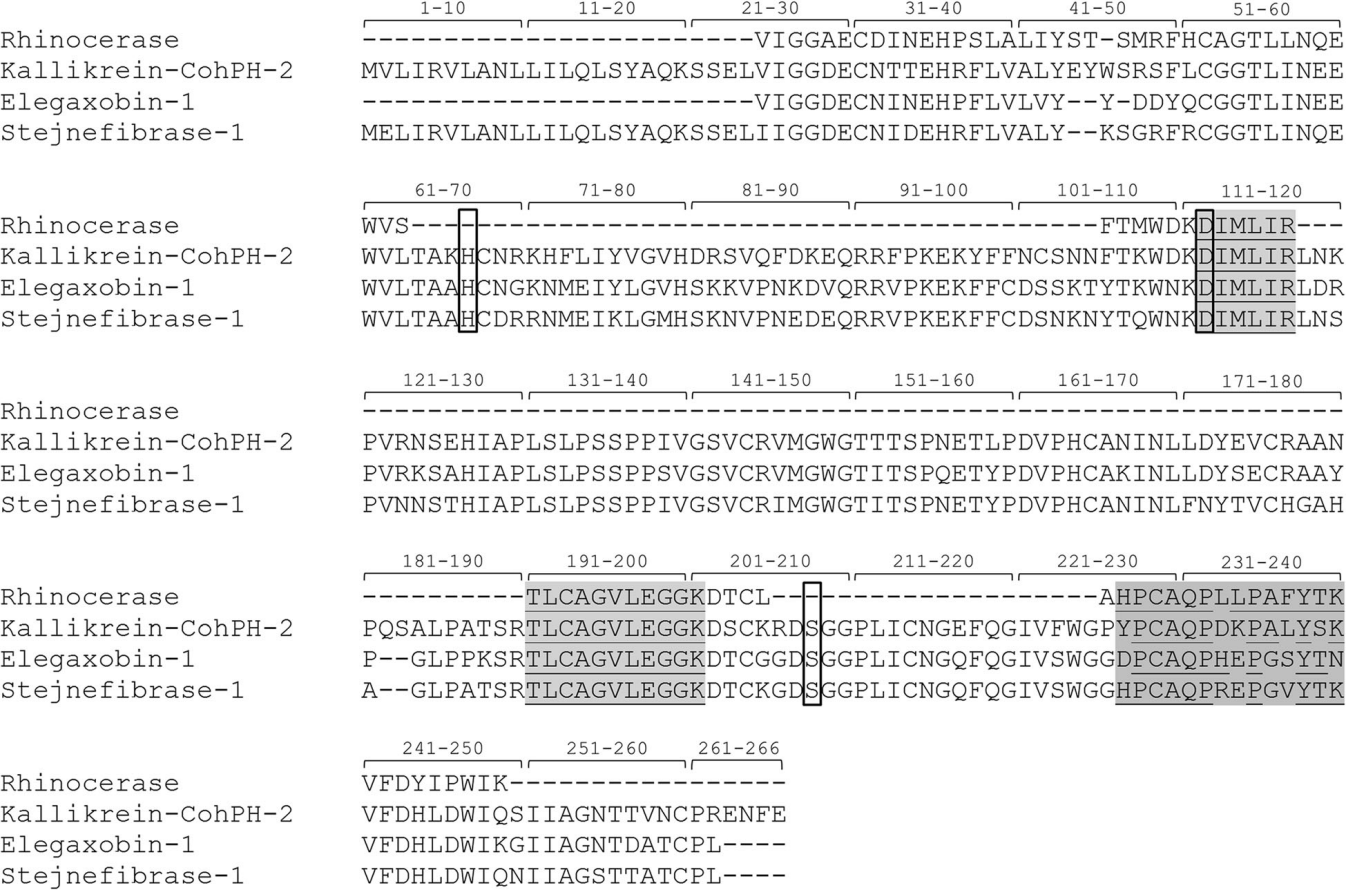
Kn-Ba identification by mass spectrometry. The three Kn-Ba peptide sequences obtained by MS/MS analysis were aligned with four known serine proteases sequences available in the UniProtKB database: rhinocerase (P86497), kallikrein-CohPH-2 (JAA98009), elegaxobin-1 (P84788) and stejnefibrinase-1 (Q8AY80). The sequences identified by de novo peptide sequencing are highlighted in gray. The residues with 100% identity are underlined. Catalytic triad is in the open box

Mass spectrometry analysis identified three peptide sequences with high confidence (Additional file 3). The sequence DIMLIR is conserved in several serine proteases from snake venoms, but only seven serine proteases described so far presented the second fragment, TLCAGVLEGGK, in the UniProtKB database. One of the hits that matched was the serine protease rhinocerase from *Bitis gabonica rhinoceros* (P86497). The other proteins presenting the fragment TLCAGVLEGGK are also serine proteases present in the venoms of two snake species, *Trimeresurus* sp. (P84788 and Q8AY80) and *Crotalus oreganus helleri* (JAA98009). Although four serine proteases from *Trimeresurus stejnegeri* present these two peptides, only Stejnefibrase-1 (Q8AY80) is shown in Fig. 3, as the other three molecules (Q8AY78; Q71QJ4 and Q71QI1) are very similar and are probably isoforms from the cited protease. Besides these two conserved peptide sequences, MS/MS analysis also revealed one exclusive Kn-Ba fragment: HPCAQPHLPAFYTK (Fig. 3).

### Kn-Ba neutralization by specific antivenom

**Fig. 4:**
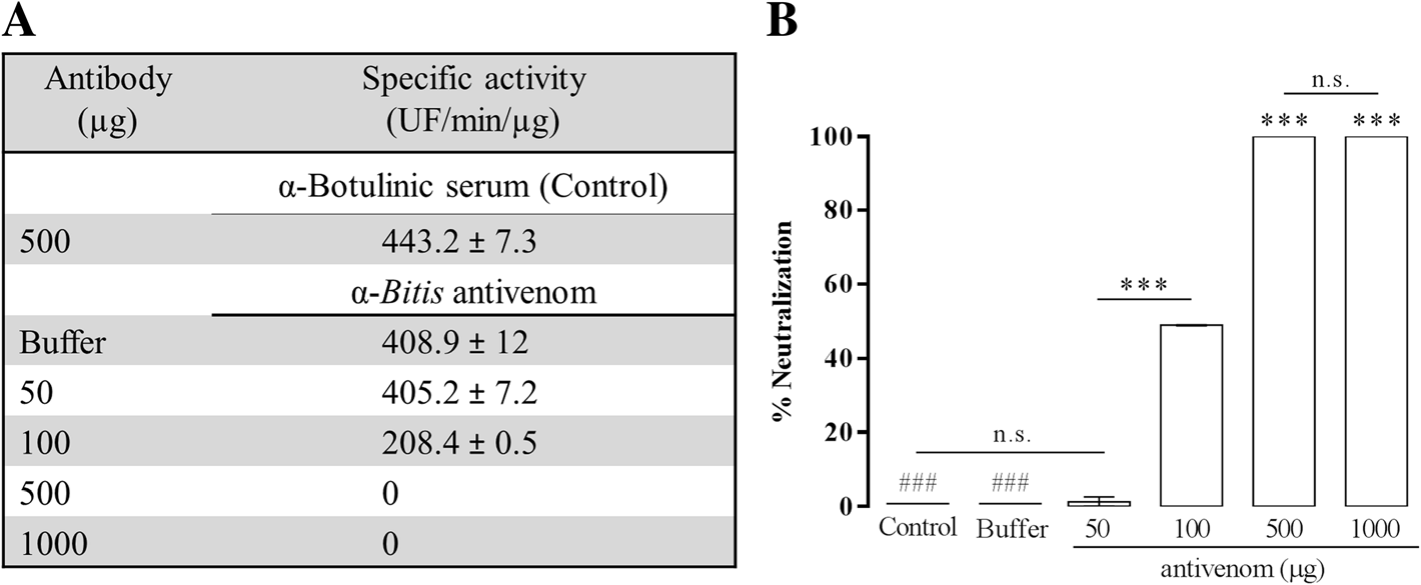
Kn-Ba neutralization by specific antivenom produced by the Butantan Institute. **a** The inhibition of Kn-Ba proteolytic activity, pretreated or not with α-Botulinic serum and α-*Bitis-arietans* antivenom, was performed upon Abz-FRSSR-EDDnp FRET substrate. **b** The percentage of neutralization was carried out and the assays were carried out in quadruplicate. Results were expressed as specific activity (UF/min/μg) ± SEM and analyzed statistically using One-Way ANOVA test followed by Tukey HSD post-hoc tests (*p < 0.05). (*) differences between samples and (#) differences between samples and controls. The hydrolysis of substrate was monitored in the spectrophotometer FLUOstar® Omega (BMG Labtech, HE, Germany; λ_EM_ 420 nm and λ_EX_ 320 nm); n.s. = not significant

The potential of experimental horse α-Ba antivenom, produced at the Butantan Institute, for neutralizing Kn-Ba proteolytic activity upon FRET substrate, was evaluated. For this, Kn-Ba (0.1 μg) was pretreated for 30 min with 50, 100, 500 or 1000 μg of α-Ba antivenom or with 500 μg of F(ab’)_2_ fragments against botulinic toxin, used as a negative control. Specific α-Ba antivenom completely neutralized Kn-Ba activity upon FRET substrate, whereas approximately 50% of the activity was neutralized by 100 μg of antivenom. In contrast, no neutralization was achieved when the purified toxin was pretreated with α-botulinic serum (Fig. 4). The neutralization of venom was similarly carried out, and the same result was observed (data not shown).

### Human fibrinogen cleavage

**Fig. 5:**
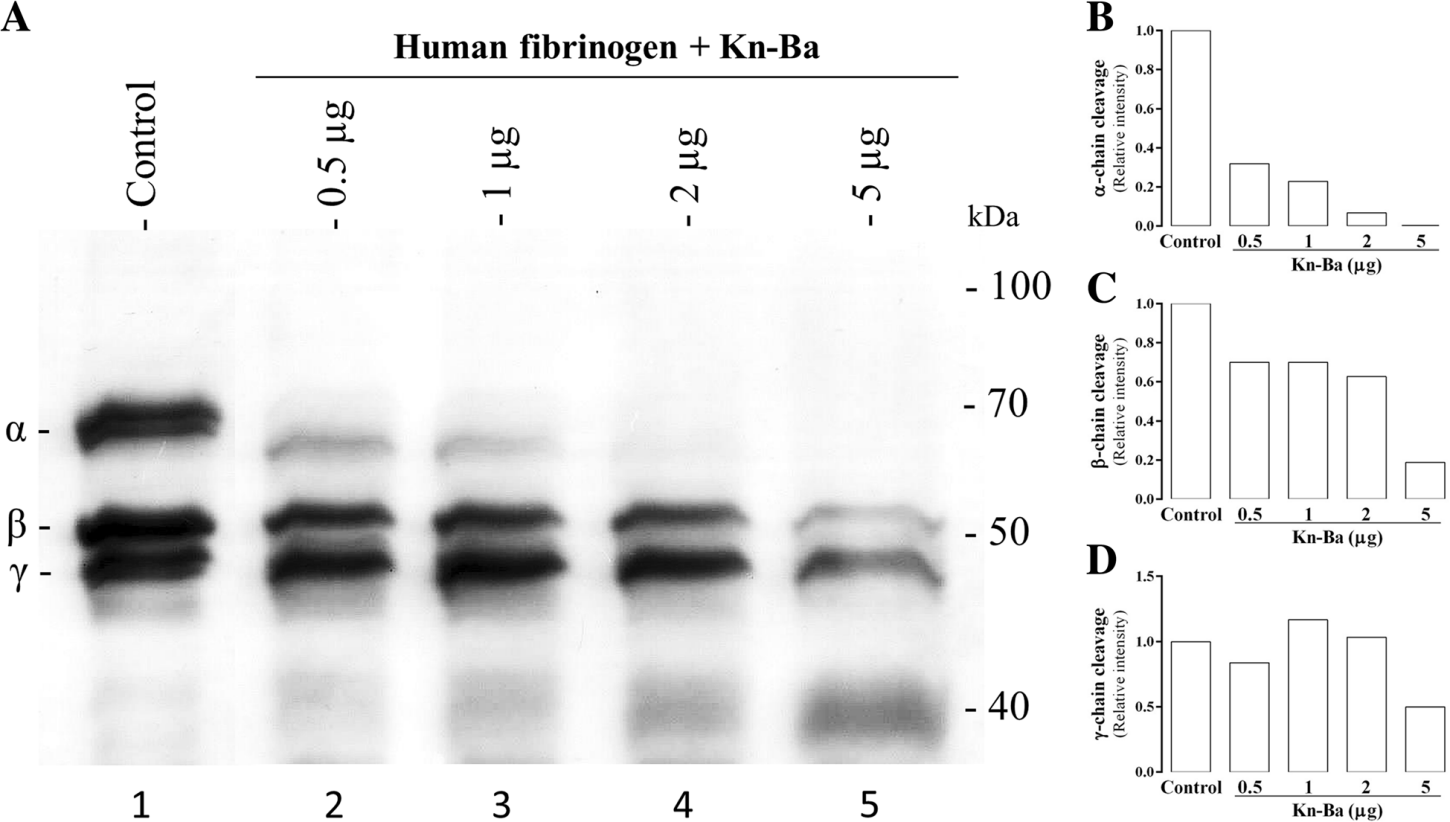
Human fibrinogen cleavage. **a** Coomassie-stained-SDS-PAGE (10%) analysis of the human fibrinogen pre-incubated with 0.5–5 μg of Kn-Ba. The relative intensities of the bands were estimated by densitometry: **b** α-chain, **c** β-chain, and (**d**) γ-chain cleavage

Kn-Ba was able to efficiently cleave α and β chains of human fibrinogen (Fig. 5). The α chain cleavage was clearly observed by incubation with the lowest amount of Kn-Ba used (0.5 μg), and was almost totally consumed when 2 μg of toxin was used. Meanwhile, more expressive β chain cleavage was observed when 5 μg of Kn-Ba was added. Based on densitometry measurements, when fibrinogen was pretreated with 5 μg of Kn-Ba, it was also possible to detect the γ chain cleavage.

### Kinin release analysis

**Fig. 6:**
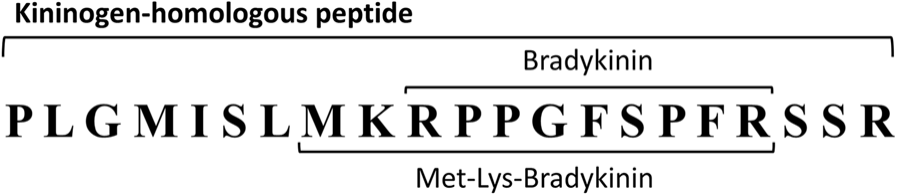
Release of biologically active kinin peptides. The kininogen-homologous peptide denominated KNBK (PLGMISLMKRPPGFSPFRSSR) was incubated with 0.2 μg of Kn-Ba in Tris-NaCl buffer (pH 7.4) at 37 °C for 3 h. The hydrolysis fragments were obtained by Zip-Tip and analyzed by mass spectrometry

After incubating the KNBK with Kn-Ba, the peptides were extracted from the solution by Zip-Tip. After eluting, peptide fragments were dried and analyzed by mass spectrometry, and two known bioactive kinins were found: Met-Lys-bradykinin and bradykinin (Fig. 6). The complete results of the MS/MS sequencing of both kinins can be found in the Additional file 4.

## Discussion

The global morbidity and mortality indices caused by snakebites are alarming, particularly in developing countries [1]. The puff adder, *B. arietans*, not only is endemic to sub-Saharan Africa, Morocco and parts of the Middle East but also is the principle species responsible for snakebite accidents in these regions [22, 36, 48]. However, despite the high number of life-threatening accidents involving *B. arietans* [1, 49], the mechanisms and toxic components of this venom which cause harm remain poorly understood. Efforts to understand how purified venom toxins act are an essential step for changing the current scenario, as new information appears promising for improving treatment. Given this need, the present study aimed to accomplish the purification and partial in vitro characterization of a serine protease from the *B. arietans* venom, since this venom presented serine protease activity, according to previous proteomic [3] and functional [50] studies. In addition, based on the hemorrhaging and hypotension presented by the victims of envenomation, fibrinogen and a peptide homologous to human kininogen were employed for the preliminary biochemical characterization of this SVSP, denominated herein as Kn-Ba.

In the current study, Kn-Ba, a serine protease with kinin-releasing and human fibrinogen-cleaving activities, was purified to homogeneity from the *B. arietans* venom through two steps of molecular exclusion chromatography. It is important to clarify that the metalloprotease activity present in the *B. arietans* venom is also capable of degrading the α and β chains of fibrinogen [50], and that based on this property we named this serine protease as Kn-Ba, because kinin-releasing activity is mainly attributed to serineproteases.

Kn-Ba, which has a molecular mass of around 33 kDa, showed proteolytic activity totally inhibited by PMSF. The peptides derived from Kn-Ba were sequenced and aligned with known serine proteases from *B. g. rhinoceros*, *Trimeresurus* sp. and *Crotalus oreganus helleri* snake venoms, presenting 100% amino acid sequence identity with them. By contrast, the third identified peptide, HPCAQPHLPAFYTK, is particular to Kn-Ba, thus indicating a novel SVSP.

Until now a vast number of snake TLEs have been described [7, 51]. However, most TLEs do not activate other coagulation cascade molecules, such as factor XIII (FXIII), an important zymogen activated by thrombin, leading to the formation of a friable fibrin-clot, which is easily removed from the circulation allowing their clinical use as a defibrinogenating agent [52, 53]. Moreover, diverse TLEs showed different secondary proteolytic sites in fibrinogen molecules, which do not always lead to clot formation. For example, rhinocerase and stejnefibrases are capable of degrading α and β chains of fibrinogen, although they are incapable of inducing platelet aggregation [13, 54]. In contrast, elegaxobin, a thrombin-like enzyme from *Trimeresurus elegans*, can cleave and clot rabbit fibrinogen [11]. Herein we showed that Kn-Ba is also capable of completely cleaving α and β chains of human fibrinogen, and that despite the fact that the releasing of fibrinopeptides and fibrinogen-clot activity by Kn-Ba remain unconfirmed, this fibrinogen-cleaving activity indicated its possible role in the hemostatic disturbances presented by envenomed victims.

Despite the limited information, it is known that envenoming by *B. arietans* results in intense hypotension for victims [22, 36]. Some studies have connected the hypotension presented by the victims to substantial bleeding in the bitten limb [55], but deaths have been reported in patients with circulatory failure without significant blood loss [22]. Thus, a direct action of the venom may be responsible for the hypotension induced by envenomation from the *Bitis* genus.

In the present report, we show that Kn-Ba releases bradykinin and, interestingly, Met-Lys-bradykinin from the kininogen-homologous peptide, which could be closely linked to the hypotension presented by victims of envenomation by *B. arietans* [22, 36].

Since Rocha and Silva and collaborators [56] described bradykinin, several kinin-releasing enzymes from snake venoms have been characterized [13, 14, 16, 17, 57]. However, kinins admittedly released by serinoproteases of animal venoms are bradykinin (BK) and kallidin (Lys-BK); to the best of our knowledge, this is the first report of the release of Met-Lys-BK by a snake venom toxin. Met-Lys-BK is considered an uncommon kinin and displays affinity for the B1 and B2 receptors, being actually equivalent to BK in terms of effective biological activity [58].

Recently, several BPPs, also known as PROs (proline-rich oligopeptides), from puff adder venom, were described [35]. These peptides can inhibit the ACE or positively modulate the catalytic activity of argininosuccinate synthase (AsS), and cause an in vivo hypotensive effect [59, 60]. Thus, we hypothesized that Kn-Ba may act synergistically with BPPs causing recurrent hypotension in cases of *B. arietans* envenoming.

Another important aspect of the present article are the studies by the Butantan Institute on specific α-*Bitis arietans* antivenom. Until now, the most commonly used and recommended treatment for snakebites is antivenom therapy, which has been improving year by year [38, 61–63]. Very importantly, this present study shows the high efficacy of the experimental anti-*B. arietans* antivenom, produced by the Butantan Institute, in neutralizing Kn-Ba, a potent serine protease from *B. arietans* venom. This antivenom presented elevated cross-recognition of proteins from *Bitis nasicornis* and *B. rhinoceros*, and furthermore was able in promote in vivo protection [38]. Based on the primary sequence homology between all serine proteases and the neutralization results shown herein, it is possible that the experimental anti-*B. arietans* antivenom is effective at blocking the SVSP activity, at least in all venoms from the *Bitis* genus.

## Conclusions

To conclude, this study described the purification and characterization of a novel bifunctional serine protease, from *B. arietans* venom, that acts upon human fibrinogen and presents kinin-releasing activity in in vitro studies. Thus, it is possible that only one serine protease is in part responsible for two important symptoms of *B. arietans* victims. Moreover, a deeper understanding of Kn-Ba mechanisms and functions may lead to new insights in clinical studies that investigate the potential of this toxin for treating human hemostatic disorders.

## Additional files

Additional file 1: Molecular exclusion chromatography of the *Bitis arietans* venom. **(A)** Twenty milligrams of freeze-dried venom was subjected to molecular exclusion chromatography on a Superose 12 HR 10/30 column, equilibrated and eluted with ammonium acetate 50 mM in a climate-controlled room (22 ± 2 °C). Samples were collected at a 0.4 ml/min flow rate, and their protein content was monitored by recording the absorbance at 280 nm in a UPC-900 monitor. **(B)** Electrophoretic profile under non-reducing conditions of the peaks obtained by molecular exclusion chromatography. (TIF 926 kb)

Additional file 2: Screening of serine-protease containing peaks. **(A)** The proteolytic activity of the pooled active peaks obtained from the first molecular exclusion chromatography upon Abz-FRSSR-EDDnp FRET substrate. The percentage of inhibition of **(B)** Fraction 2 (F2) and **(C)** Fraction 3 (F3) using EDTA, PMSF and PHE was duly performed. These assays were performed in quadruplicate. Results were expressed as specific activity (UF/min/μg) ± SEM and analyzed statistically using One-Way ANOVA test followed by Tukey HSD post-hoc tests (*p < 0.05). (TIF 118 kb)

Additional file 3: Amino acid sequence of Kn-Ba peptides. Amino acid identification and molecular mass of Kn-Ba-derived peptides: **(A)** DIMLIR, **(B)** TLCAGVLEGGK and **(C)** HPCAQPHLPAFYTK, as determined by mass spectrometry. (TIF 1649 kb)

Additional file 4: Amino-acid sequence of peptides derived from kininogen-homologous peptide cleavage upon Kn-Ba treatment. Amino-acid identification and molecular mass of KNBK-(PLGMISLMKRPPGFSPFRSSR)-derived peptides: **(A)** RPPGFSPFR and **(B)** MKRPPGFSPFR as determined by mass spectrometry. (TIF 1555 kb)

## Abbreviations

Abz: *O*-aminobenzoic acid
BCA: Bicinchoninic acid
Da: Dalton
EDDnp: *N*-(2,4-dinitrophenyl)-ethylenediamine
EDTA: Ethylene diamine tetracetic acid
F(ab’)_2_: Fragment antigen binding of antibodies generated by pepsin digestion
HPLC: High performance liquid chromatography
LTQ: Linear trap quadropole
m/z: Mass-to-charge ratio
MS: Mass spectrometric or mass spectrometry
MS/MS: Tandem mass spectrometry
nLC: Nano*-*liquid chromatography
PHE: 1,10-phenanthroline
PMSF: Phenylmethylsulfonyl fluoride
SDS-PAGE: Sodium dodecyl sulfate-polyacrylamide gel electrophoresis
TFA: Trifluoroacetic acid
U-E/mL: ELISA units/mL
WHO: World Health Organization
