# Supplementary figures and images for: Kn-Ba: a novel serine protease isolated from Bitis arietans snake venom with fibrinogenolytic and kinin-releasing activities
Source: J Venom Anim Toxins Incl Trop Dis. 2018 Dec 13;24:38. doi: 10.1186/s40409-018-0176-5 (PMC6293559; doi:10.1186/s40409-018-0176-5)

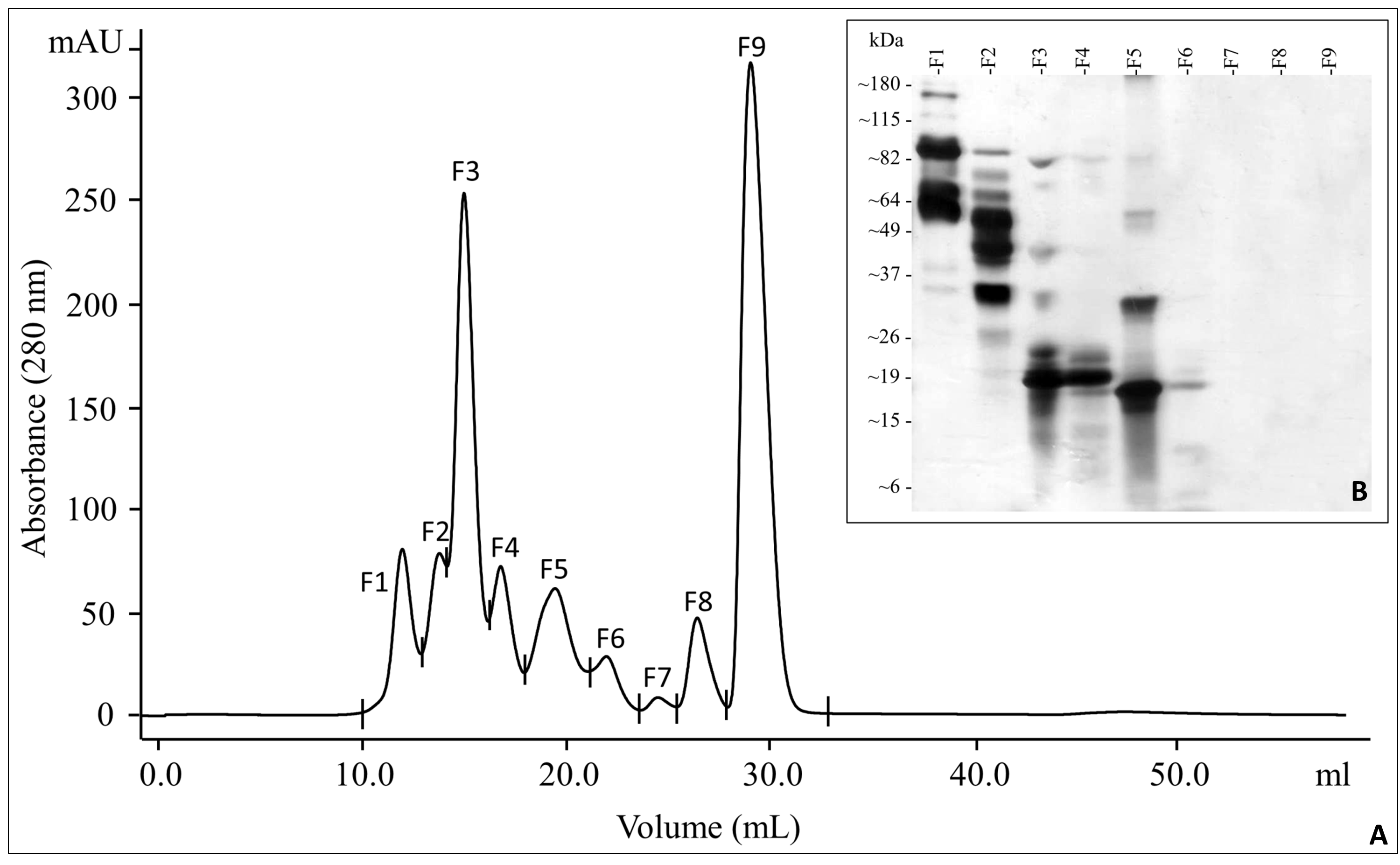

Supplement: Supplementary file 1 — Molecular exclusion chromatography of the Bitis arietans venom. (A) Twenty milligrams of freeze-dried venom was subjected to molecular exclusion chromatography on a Superose 12 HR 10/30 column, equilibrated and eluted with ammonium acetate 50 mM in a climate-controlled room (22 ± 2 °C). Samples were collected at a 0.4 ml/min flow rate, and their protein content was monitored by recording the absorbance at 280 nm in a UPC-900 monitor. (B) Electrophoretic profile under non-reducing conditions of the peaks obtained by molecular exclusion chromatography. (TIF 926 kb) [file 40409_2018_176_MOESM1_ESM.tif]

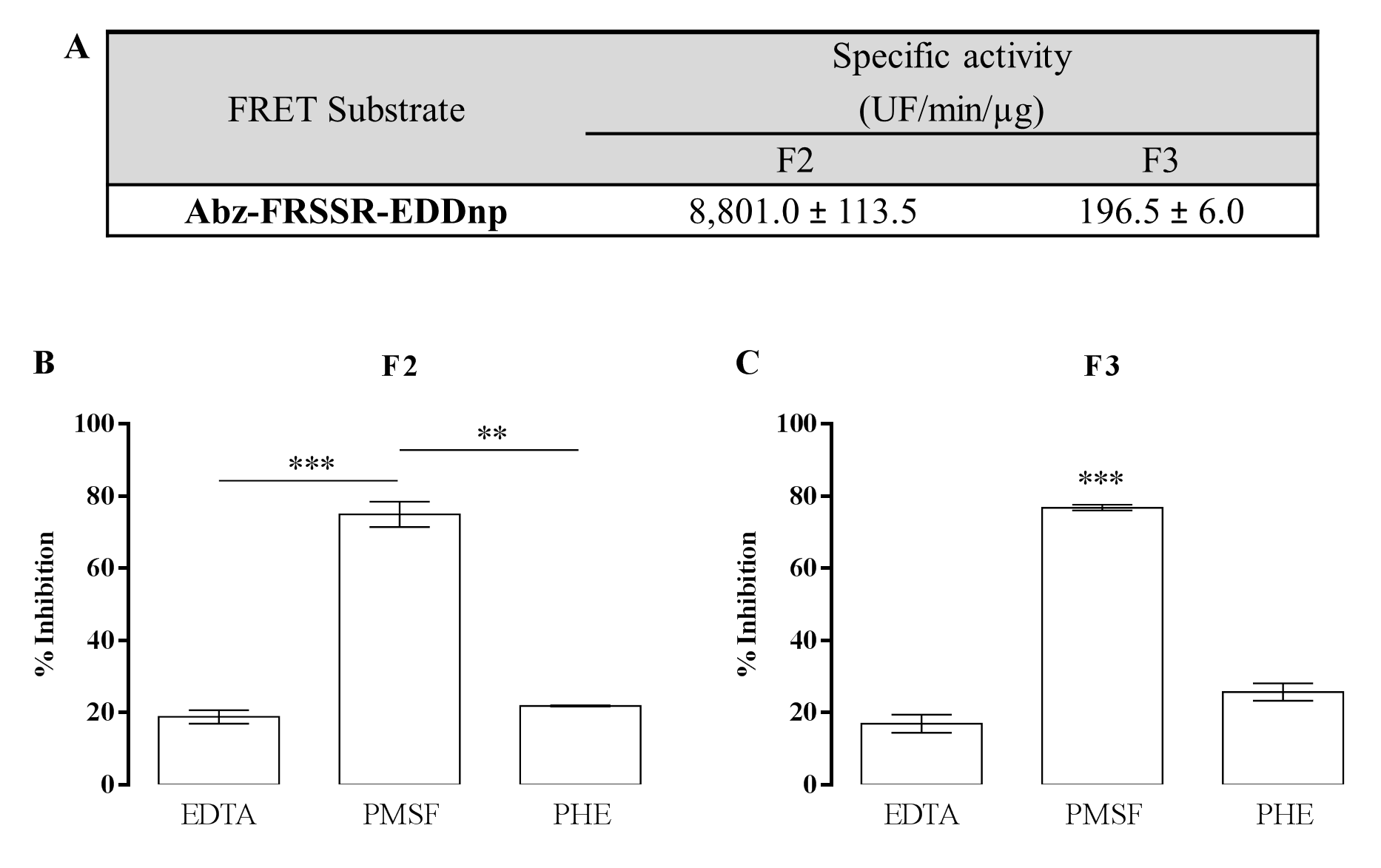

Supplement: Supplementary file 2 — Screening of serine-protease containing peaks. (A) The proteolytic activity of the pooled active peaks obtained from the first molecular exclusion chromatography upon Abz-FRSSR-EDDnp FRET substrate. The percentage of inhibition of (B) Fraction 2 (F2) and (C) Fraction 3 (F3) using EDTA, PMSF and PHE was duly performed. These assays were performed in quadruplicate. Results were expressed as specific activity (UF/min/μg) ± SEM and analyzed statistically using One-Way ANOVA test followed by Tukey HSD post-hoc tests (*p < 0.05). (TIF 118 kb) [file 40409_2018_176_MOESM2_ESM.tif]

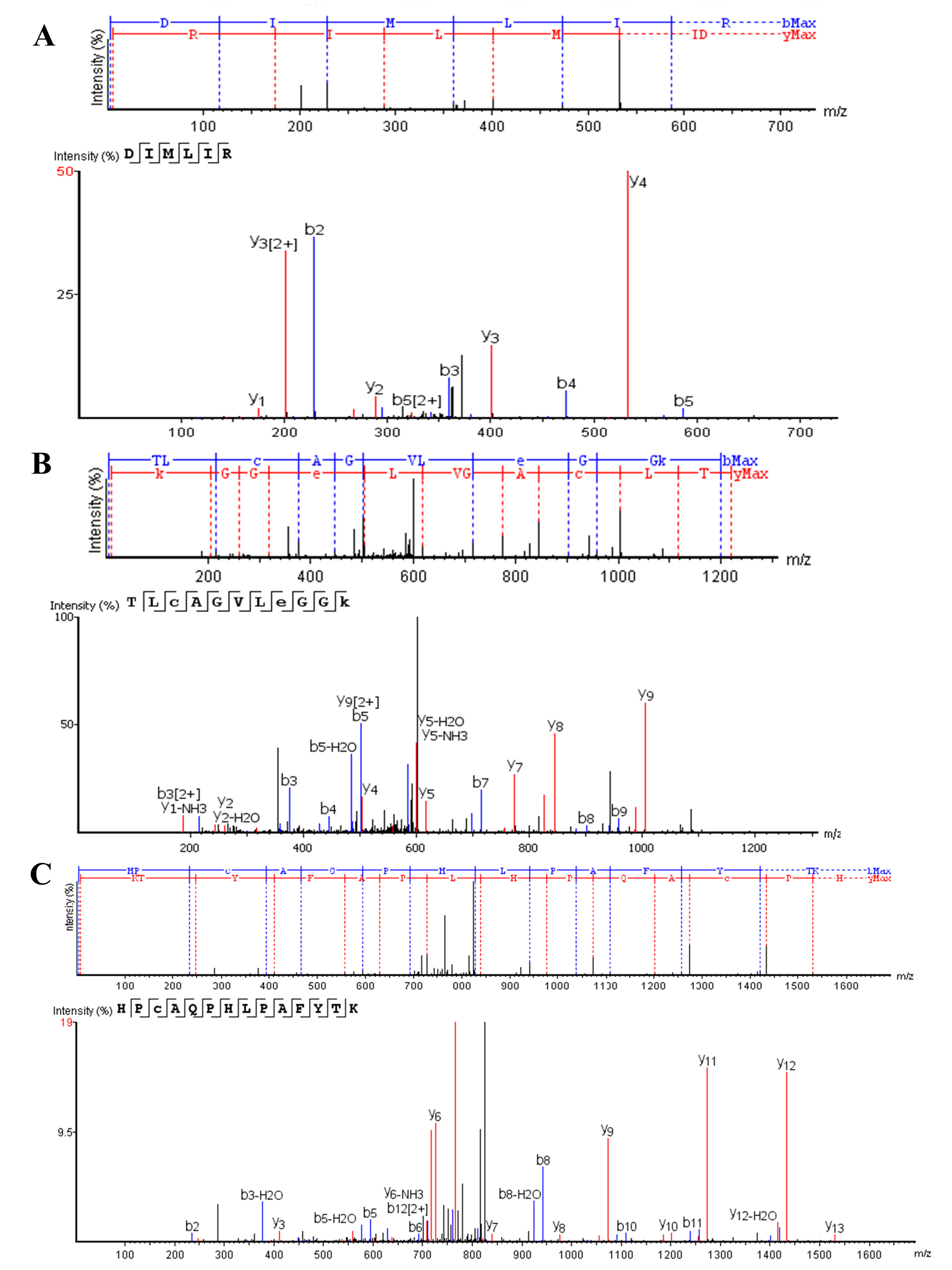

Supplement: Supplementary file 3 — Amino acid sequence of Kn-Ba peptides. Amino acid identification and molecular mass of Kn-Ba-derived peptides: (A) DIMLIR, (B) TLCAGVLEGGK and (C) HPCAQPHLPAFYTK, as determined by mass spectrometry. (TIF 1649 kb) [file 40409_2018_176_MOESM3_ESM.tif]

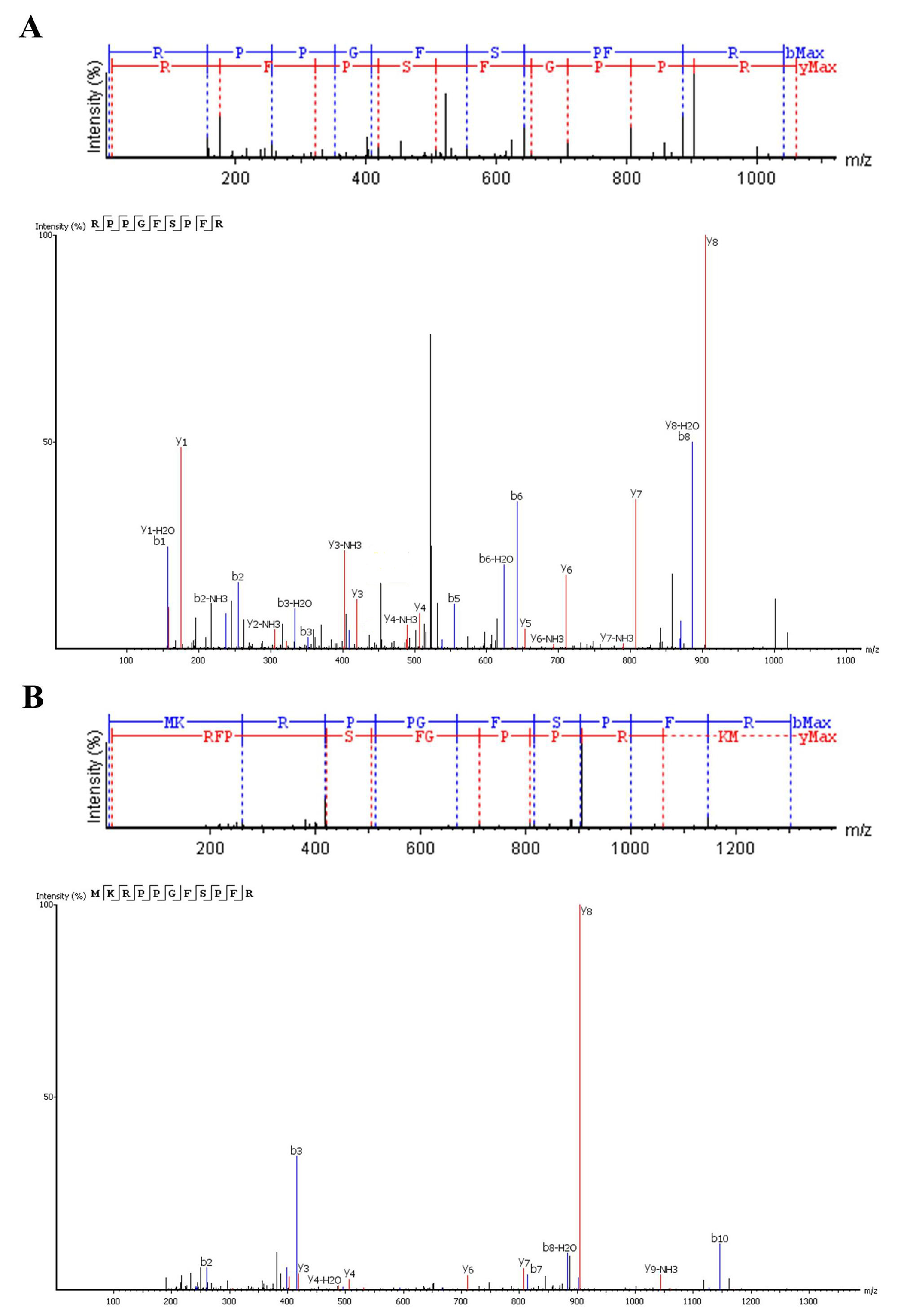

Supplement: Supplementary file 4 — Amino-acid sequence of peptides derived from kininogen-homologous peptide cleavage upon Kn-Ba treatment. Amino-acid identification and molecular mass of KNBK-(PLGMISLMKRPPGFSPFRSSR)-derived peptides: (A) RPPGFSPFR and (B) MKRPPGFSPFR as determined by mass spectrometry. (TIF 1555 kb) [file 40409_2018_176_MOESM4_ESM.tif]
